# P300 Development across the Lifespan: A Systematic Review and Meta-Analysis

**DOI:** 10.1371/journal.pone.0087347

**Published:** 2014-02-13

**Authors:** Rik van Dinteren, Martijn Arns, Marijtje L. A. Jongsma, Roy P. C. Kessels

**Affiliations:** 1 Research Institute Brainclinics, Nijmegen, The Netherlands; 2 Donders Institute for Brain, Cognition, and Behaviour, Radboud University Nijmegen, Nijmegen, The Netherlands; 3 Utrecht University, Department of Experimental Psychology, Nijmegen, The Netherlands; 4 Behavioral Science Institute, Radboud University Nijmegen, Nijmegen, The Netherlands; 5 Department of Medical Psychology, Radboud University Nijmegen Medical Centre, Nijmegen, The Netherlands; University of Rome, Italy

## Abstract

**Background:**

The P300 component of the event-related potential is a large positive waveform that can be extracted from the ongoing electroencephalogram using a two-stimuli oddball paradigm, and has been associated with cognitive information processing (e.g. memory, attention, executive function). This paper reviews the development of the auditory P300 across the lifespan.

**Methodology/Principal Findings:**

A systematic review and meta-analysis on the P300 was performed including 75 studies (n = 2,811). Scopus was searched for studies using healthy subjects and that reported means of P300 latency and amplitude measured at Pz and mean age. These findings were validated in an independent, existing cross-sectional dataset including 1,572 participants from ages 6–87. Curve-fitting procedures were applied to obtain a model of P300 development across the lifespan. In both studies logarithmic Gaussian models fitted the latency and amplitude data best. The P300 latency and amplitude follow a maturational path from childhood to adolescence, resulting in a period that marks a plateau, after which degenerative effects begin. We were able to determine ages that mark a maximum (in P300 amplitude) or trough (in P300 latency) segregating maturational from degenerative stages. We found these points of deflection occurred at different ages.

**Conclusions/Significance:**

It is hypothesized that latency and amplitude index different aspects of brain maturation. The P300 latency possibly indexes neural speed or brain efficiency. The P300 amplitude might index neural power or cognitive resources, which increase with maturation.

## Introduction

Almost half a century ago, the group of Samuel Sutton and E.R. John first described the P300 [1], a component from the event-related potential (ERP), which has been intensively investigated since then. However, despite abundant research on this component, its developmental path across the lifespan has been relatively underexposed. The present paper will review and analyze the developmental process of the auditory P300 across the lifespan employing 1) a systematic review and meta-analysis of all studies published over the last half century and 2) an independent cross-sectional dataset including 1,572 participants. The P300 developmental process is proposed to reflect development of cognitive speed and cognitive capacity, across the lifespan.

### Characteristics of the P300

**Figure 1:**
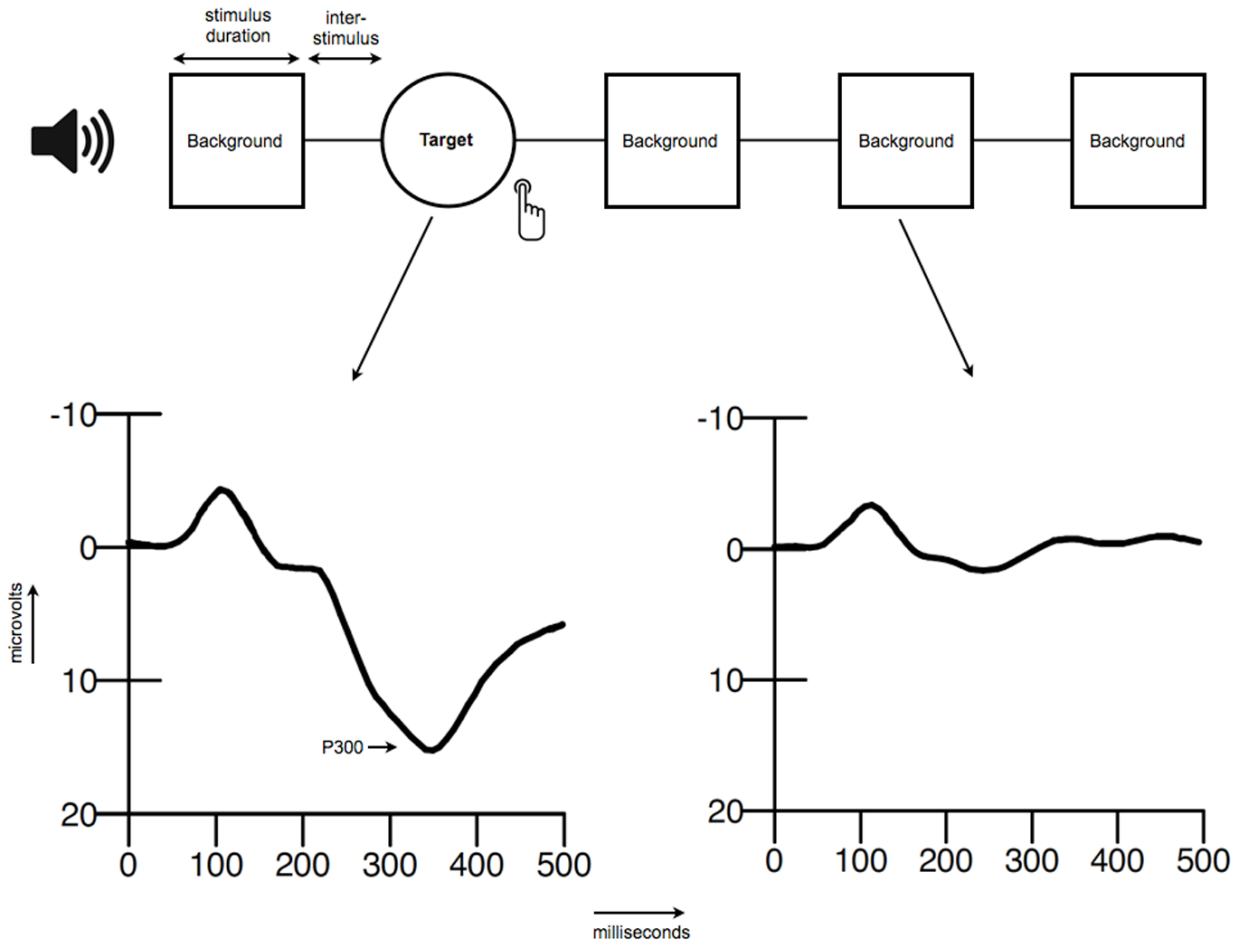
Schematic overview of the oddball paradigm and an example of an ERP.

The ERP is quantified by averaging activity in the electroencephalogram (EEG) time-locked to a specific event, for instance an auditory stimulus. This results in a waveform associated with the processing of that specific event. The ERPs found in such tasks have a characteristic waveform with clearly identifiable components, which are named after their polarity and approximate latency (i.e., P100, N100, P200, N200, P300). The P300 (also referred to as P3) component of the auditory ERP is a large positive waveform that reaches a maximum at approximately 300 milliseconds after stimulus onset (see figure 1 for an example). The amplitude is defined as the voltage difference between a pre-stimulus established baseline and the largest positive peak within a predefined latency window [2].

The P300 is commonly elicited in signal-detection tasks. A typical signal-detection paradigm is the ‘oddball’ paradigm that was first used by Ritter and Vaughan [3]. In the auditory version of this paradigm, participants are typically presented with two different tones that can be discriminated based on, for example, pitch or loudness. The different types of tones are presented with different probabilities (e.g., 20% vs. 80%). The frequent stimuli are designated as background stimuli, the infrequent stimuli as target or oddball stimuli to which the participant must respond, for example by counting or pressing a button [3]–[5]. See figure 1 for a schematic overview of the oddball.

In addition to the traditional P300 that is associated with responding to infrequent target stimuli, a slightly earlier P3 peak has been reported, which has slightly shorter latencies and a more frontally oriented topography. This component has also been labeled as *P3a* [6]–[9], and has primarily been linked to stimulus novelty and is not necessarily related to the generation of responses. This peak can be observed in, for example, a modified three-stimulus oddball paradigm including a second infrequent stimulus. Consequently, the P300 component described earlier has also been labeled *P3b*. Throughout this paper we will use the term P300 to reflect the P3b.

### P300 Theories

A central theme in P300 research is the exact nature of the involved cognitive processes underlying the P300, and several theories have been postulated in this respect. First, the stimulus-evaluation hypothesis states that the latency of the P300 component reflects the time needed for stimulus evaluation processes and is independent of the time needed for response processes [10]. However, this theory has been refuted in a more recent review [11], [12]. A more prominent hypothesis, which has its roots in Sokolov’s orienting response model and links the P300 to cognitive functioning, is the context-updating hypothesis [4], [5], [13]. This hypothesis states that the P300 (amplitude) represents brain activity related to updating a mental stimulus representation when deviant stimuli are presented. That is, the participant’s mental model of his/her environment, or context, is evaluated and updated when a relevant and deviant stimulus is presented [2], [5], [13]. Finally, as an alternative to the context-updating hypothesis, the context-closure hypothesis has emerged. This hypothesis links the P300 to deactivation processes, consequent upon the closure of a perceptual epoch. The hypothesis states that participants combine repeatedly presented stimuli in meaningful contexts. Deviating target stimuli, after a series of non-deviating background stimuli, close such a context and this closure process is reflected by the P300 [5], [14]–[16].

Although these hypotheses recognize the involvement of various cognitive processes, still, after almost 50 years of intensive research with over 12,000 publications on the P300 it has not been possible to link the P300 to a specific cognitive process. Presumably, the P300 complex is multifarious, reflecting a culmination of multiple cognitive processes. However, there is evidence that shorter P300 latencies and larger amplitudes are associated with superior information processing [4], [5], [17]–[20]. In addition to individual differences due to trait effects, the P300 is also influenced by state variables, that is, natural and induced biological factors – like body temperature, sleep quality, exercise, food intake, drugs – which are mediated by arousal levels [21]. Thus, an interaction of cognitive processes and arousal levels determine relative changes in the P300, that is, component latencies and amplitudes. The absolute P300 morphology is predominantly determined by an individual’s physiological properties, such as anatomical features of the corpus callosum [22] or skull thickness [23]. Thus despite relative changes by state variables, a person’s specific P300 morphology is a remarkably stable measure that shows little variation over recording sessions or experiments [24]. In line, P300 morphology has demonstrated a high heritability of approximately 60% [25]. The main aim of the current review is to unravel the P300’s development across the lifespan based on data obtained from both a meta-analysis and systematic review of existing papers and an independent large standardized dataset. First, a descriptive model of P300 development across the lifespan will be presented. This model will then be used to describe the development of information processing in terms of cognitive speed and resources.

### Age Effects on the P300 Latency

Research on P300 development across the lifespan has been relatively scarce. However, there is clear evidence that P300 latency decreases during the first years of life [26]–[29], whereas in older adults the parietal P300 latency increases [30]–[33]. This model describing initial maturation followed by degenerative effects of aging on latency indicates that there may be a specific age range that marks a point of deflection in P300 latency development. To our knowledge, this specific trough of the P300 latency has not been described yet.

### Age Effects on the P300 Amplitude

Findings on early developmental processes in P300 amplitude are mixed. P300 amplitudes are found to either increase during childhood or show no change [26], [27], [29], [34]. Capacity of information processing increases rapidly during early childhood, which is expected to enhance the P300 amplitudes. However, an opposing effect on amplitudes may result from an increase in skull thickness, as a thicker skull is related to smaller amplitudes [23]. Indeed, a study by Beauchamp et al. found an increasing brain-scalp distance as children age [35]. Thus, cranial development during childhood probably moderates early P300 amplitude development.

In adulthood, a decline of the parietal P300 amplitude with advancing age is commonly reported [30]–[33]. Since smaller P300 amplitudes have been associated with a decreased performance on a variety of cognitive tests indexing different aspects of information processing [5], they might thus reflect aging-related cognitive decline.

### Behavioral Task Performance

An elegant aspect of the oddball paradigm is the possibility to quantify psychophysiological measures – i.e., the P300 latency and amplitude as described above – with their consecutive behavioral measures, such as reaction times (RTs) and errors. Speed variables – like RTs – have moderate to large associations with age during adulthood [36]. Therefore, RTs have been hypothesized to be an index of aging-related cognitive decline. In general, measures of speed tend to share 75% of the age-related variance with a variety of cognitive measures [36]. Thus, directly linking behavioral measures to the analysis of P300 latency and amplitude could result in new insights into the underlying neurocognitive mechanisms of the P300 across the lifespan.

### The Present Review

First, a systematic review and meta-analysis will be performed, in order to model P300 developmental trajectories across the lifespan. The results of this review will be used to investigate effects of paradigm and sample characteristics on P300 latency and amplitude. Based on a literature review published by Polich (1996), besides age, an effect of stimulus saliency attributes, like stimulus intensity, stimulus duration and number of stimuli, is expected [5].

Second, based on an independent multi-site, cross-sectional dataset of 1,964 healthy participants aged 6–87 years – who all performed the same paradigm under standardized recording conditions and identical task procedures – an age-based model of the P300 across the lifespan will be estimated. In addition, the effects of within subject variables such as sex and education will be investigated further.

Finally, the estimated developmental trajectory of the P300 latency and amplitude will be compared to the developmental trajectory of behavioral measures such as reaction times and number of errors. Previous research has demonstrated that the P300 latency shows a significant positive correlation with reaction times [2].

## Methods

### Meta-analysis

#### Literature search

A systematic review was performed using Scopus with the search phrase “*P300 OR P3b AND oddball*”, starting on June 18^th^ 2012 until October 10^th^ 2012. The search was not conducted according to a specific review protocol. All article titles and summaries were scanned for selection criteria. When the summary provided insufficient information, the methods section of the article was read. The following criteria were used to assess eligibility of articles for the meta-analysis:

- The study had to report data on healthy participants not diagnosed with any neurological, psychiatric or other disorder which have a significant impact on the P300. When such information was not reported, a study could not be included. In studies that used a clinical population, only data from the healthy control participants were included.
- The study had to report a mean age and a mean P300 latency and/or P300 amplitude for the healthy participants. These measures had to be reported quantitatively. Studies that reported only P300 data in graphs were excluded.
- Studies with fewer than 15 participants in total were excluded in order to prevent Type-I errors [5], [37], [38].
- Studies had to employ an auditory, active (meaning a response required from the participant on the oddball), two-stimulus (auditory stimuli differing in frequency) oddball paradigm. These criteria were based on relevant parameters mentioned in Polich (1996). The current meta-analysis focused on (binaurally) auditory paradigms since this is in accordance with the paradigm used in the cross-sectional data set.
- P300 data for the Pz electrode site had to be available.
- Study results had to be available in English.
- Meta reviews and overlapping data sets (i.e., multiple papers on the same sample) were excluded.
- Studies using the BRID database xREF: www.brainnet.net) were excluded, since data from this database were included in part 2 of this study.

**Figure 2:**
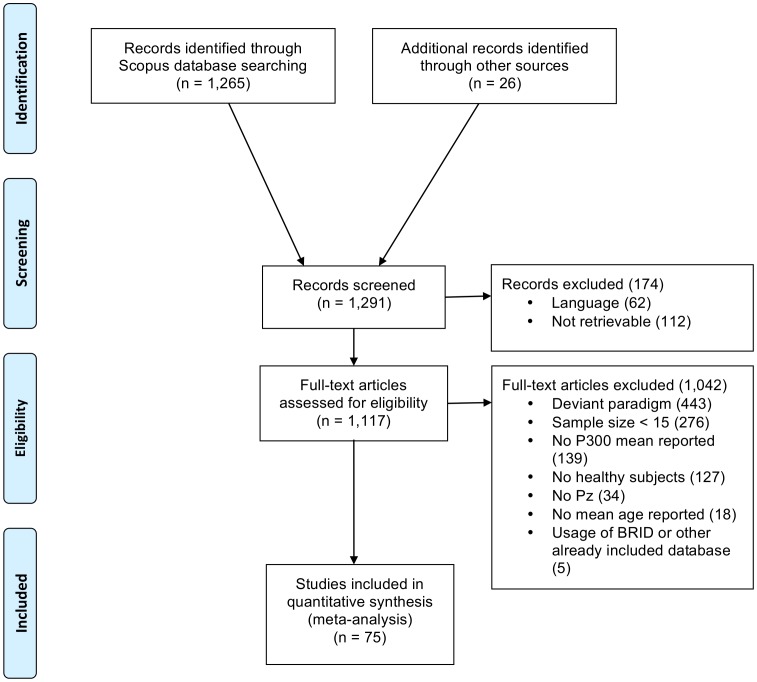
Flowchart depicting the number of exclusions per exclusion rationale in the literature selection.

The literature search resulted in a total of 1,265 studies. The literature review by Polich (1996) was checked manually for additional references that fulfilled the search selection criteria since it served as a basis for the current meta-analysis [5]. This yielded 26 additional studies resulting in a total of 1,291 studies. Figure 2 presents a flowchart depicting the number of studies that were excluded and the reasons for exclusion. Exclusion rationale was scored only once per study, and the main reason was noted. Therefore, some of the studies in figure 2 may meet multiple exclusion criteria.

#### Data extraction

The resulting 75 studies that were used in the meta-analysis are listed in table S1 with study details. The data entered per study are listed below:

1. *Year* was defined as the year of publication
2. C*ontinent* (if not clear, the first author’s affiliation was used)
3. *Number of participants* was recorded as detailed as possible. So when data was divided over several age groups the data for each group were recorded. This applies to all data mentioned below
4. *Percentage of males* (if available)
5. *Mean age*
6. *Mean P300 latency* in milliseconds (and standard deviation if available)
7. *Mean P300 amplitude* in microvolts (and standard deviation if available).

In addition, recording parameters were also extracted. The parameter selection was based on Polich (1996) and included 1) eyes open/eyes closed if reported; 2) response type (press or count); 3) stimulus duration; 4) stimulus intensity; 5) absolute tone frequency difference between target and background. Additionally, 6) target probability; 7) total number of stimuli and 8) inter-stimulus interval were scored.

After the initial selection of appropriate studies, efforts were made to retrieve missing data by contacting the authors. All data were entered in a single spreadsheet using Microsoft Excel 2011. This spreadsheet was fully double checked for transcription errors by a second independent rater.

#### Outlier removal

One study reported a mean amplitude that was four standard deviations from the general mean and greatly affected average amplitude data [39]. The authors from this study mentioned several factors that may have attributed to the high amplitude scores they found, namely, the use of a low target probability, large inter-stimulus intervals, morning assessments and the use of a group of young adults [39]. Therefore, this study was considered an outlier and excluded from the analyses.

#### Statistical analysis

Visual inspection of the graphed latency data revealed a suspected inversed Gaussian pattern for the latency data. The latency data were transformed by inversing the scores and adding a constant (i.e., 600) in order to fit a model to the data. Transformation of the amplitude data was not necessary.

The data were entered in Graphpad Prism 6.0. Every datapoint consisted of a mean age and mean P300 latency or amplitude value. Prism has the possibility to include standard deviation and sample size in the calculations and these measures were entered when they were available. Several models were fitted to the data including a normal Gaussian, a logarithmic Gaussian and a straight line. The Gaussian models are described by three parameters; its center, width and amplitude. Center is the *x* value at the peak of the distribution; width is a measure of the width of the distribution expressed in the same units as *x;* amplitude is the height of the center of the distribution expressed in *y* units (www.graphpad.com). The model’s amplitude is referred to as *height* to avoid confusion with the P300 amplitude.

In addition, one-way ANOVAs were performed to investigate the effect of *eyes open/closed* and *response type*. The other predictors were investigated using regression analysis. These predictors were entered in two blocks. The first block contained predictors based on the meta-analysis by Polich (1996) and included: *percentage of males, number of stimuli, and stimulus duration.* The second block included: *target probability, stimulus loudness, frequency difference between target and background tone, inter-stimulus interval*. Then, the regression model was refined by entering only those predictors that showed an effect with a significance of p<0.10. For the final regression model a significance level of p<.01 was used.

### Cross-sectional dataset

#### Ethics statement

All participants gave written informed consent. Local independent review board approval was obtained for all clinics.

#### Participants

Normative data were extracted from the Brain Resource International Database (BRID) resulting in a dataset of 1,964 healthy participants. This database contains data from multiple laboratories (New York, Rhode Island, Nijmegen, London, Adelaide, and Sydney) using standardized data acquisition techniques (identical amplifiers, standardization of other hardware, audio calibration, paradigm details, software acquisition, and task instructions). Inter-lab reliability and test-retest reliability measures are high and have been reported elsewhere [24], .

Participants were aged 6 to 87 (mean  = 28.38±20.08). The sample consisted of 992 male and 972 female participants. Education scores varied from 1 to the maximum possible score of 18 years of education (mean  = 10.89±4.46). Database exclusion criteria included a personal or family history of mental illness, brain injury, neurological disorder, serious medical condition, drug/alcohol addiction, first-degree relative with bipolar disorder, schizophrenia, or genetic disorder. Participants were required to refrain from caffeine and smoking for at least 2 hours and alcohol for at least 6 hours prior to testing.

Six participants with error rates of 33% or higher on false positive errors (button press on a background stimulus) or false negative errors (no button press on a target stimulus) were considered unreliable and removed from the dataset (as an indication they have not understood the task instructions appropriately). The remaining 1,958 participants were matched for age and sex by random selection, resulting in a further exclusion of 386 participants. The resulting dataset consisted of 786 males and 786 females matched for age (mean  = 27,17±19.16; range  = 6–87 years).

#### Electroencephalographic data acquisition

EEG and ERP recordings were performed using a standardized methodology and platform (Brain Resource Ltd., Australia). Details of this procedure have been published elsewhere [24], [42].

In summary, participants were seated in a sound and light attenuated room, controlled at an ambient temperature of 22°C. EEG data were acquired from 26 channels: Fp1, Fp2, F7, F3, Fz, F4, F8, FC3, FCz, FC4, T3, C3, Cz, C4, T4, CP3, CPz, CP4, T5, P3, Pz, P4, T6, O1, Oz and O2 (Compumedics Quikcap and NuAmps amplifier; 10–20 electrode international system). Data were offline referenced to averaged mastoids with a ground at Fpz. Horizontal eye movements were recorded with electrodes placed 1.5 cm lateral to the outer canthus of each eye (bipolar). Vertical eye movements were recorded with electrodes placed 3 mm above the middle of the left eyebrow and 1.5 cm below the middle of the left bottom eyelid. Skin resistance was <5 kOhms for all electrodes. A continuous acquisition system was employed and EEG data were EOG corrected offline [43]. The sampling rate of all channels was 500 Hz. A high cut-off filter at 100 Hz was employed prior to digitization. P300 latency and amplitude were quantified at Pz.

#### Auditory oddball

The oddball paradigm consisted of a quasi-random sequence of 280 frequent background tones (500 Hz) and 60 infrequent target (1000 Hz) tones. Two targets could not appear consecutively. All stimuli (50 ms; 5 ms rise and fall time) were presented binaurally at a volume of 75dB SPL with an inter-stimulus interval of 1000 ms. Participants were instructed to press two buttons simultaneously (one for each index finger) when they heard a target tone and to ignore the background tones. Speed and accuracy of response were both equally stressed in the instructions. Before the actual test they were presented with a brief practice run to clarify the distinction between the two tones.

#### Statistical analysis

Effects of age, sex and education on P300 latency and amplitude were investigated. Using Graphpad Prism 6.0, non-linear regression analyses by means of a curve fit were carried out for both latency and amplitude across the lifespan. Three different models were compared by an extra sum-of-squares F test. In addition, a one-way ANOVA was used to investigate sex effects. The significance level was set at p<.01 due to the large sample size. Also, separate curves were determined for males and females and were statistically compared. Education effects were investigated in a subgroup of adults using regression analysis.

Effects of age, sex and education on behavioral measures were also studied. The procedure for reaction times was identical to the latency analysis. Additionally, correlations between reaction times and P300 latency and amplitude were investigated. Lastly, correlations between the number of errors, and age, latency, amplitude and reaction time were investigated.

## Results

### Meta-analysis

#### Study characteristics

There were 75 studies selected for the meta-analysis. These were published between 1987 and 2012. All participants together (n = 2,811) had a mean age of 33.3 ranging from 4 to 95 years. The overall mean P300 latency was 316.5 milliseconds (range: 290.0–447.5) and the overall mean P300 amplitude was 10.4 microvolts (range: 2.6–37.7).

#### Psychophysiology

**Figure 3:**
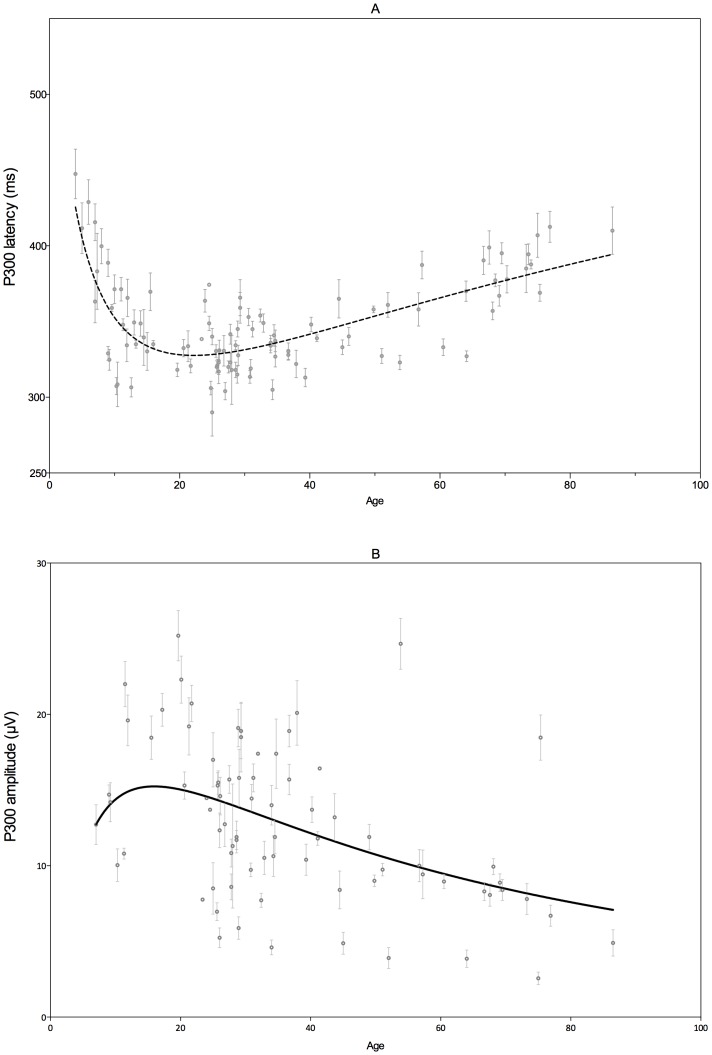
P300 latency and amplitude trajectories across the lifespan as obtained from the meta-analysis. Dots represent (subgroups from a) study. Error bars represent SEM.

Figure 3a shows the P300 latency across the lifespan as obtained from the meta-analysis. A logarithmic Gaussian model was the best fit when compared to a (normal) Gaussian model (F(1,2511) = 76.90; p<.0001) or a linear model (F(1,1569) = 330.6; p<.0001) and accounted for approximately 19% of the variance. The model reveals a trajectory in which the P300 latency decreases during childhood, reaching a trough around an age of 22 years, followed by a slow increase for the rest of the lifespan.

The P300 amplitude trajectory is shown in figure 3b. For amplitudes a logarithmic Gaussian model was also the best fit when compared to a (normal) Gaussian model (F(1,2146) = 121.6; p<.0001) or a linear model (F(1,2146) = 24.39; p<.0001). The model accounted for 9% of the variance. The maximum P300 amplitude was estimated at an age of 16 years.

#### Paradigm parameters and within-subject characteristics

Effects of paradigm parameters and within-subject characteristics were investigated in a subgroup in which age effects on latency and amplitude were linear so that regression analysis was possible. This subgroup was defined by the high end of the 95% confidence interval for the age center value, respectively for latency (age 25.5) and amplitude (age 22.5), until the age of 65.

For latency, one-way ANOVAs for *eyes open/closed* (F(1,18) = 1.084) and *response type* (F(1,48) = 2.478) were not statistically significant. These variables were therefore not included in the regression analysis. The other predictors were entered in a regression model in two blocks. Block 1 of the regression model revealed no significant predictors apart from *age*. Block 2 revealed *target probability*, *stimulus duration* and *inter-stimulus interval* as possibly significant predictors. These were entered in a final regression model that was not significant.

For amplitude, one-way ANOVAs for *eyes open/closed* (F(1,21) = 2.112) and *response type* (F(1,54) = 0.011) were not significant. These variables were therefore not included in the regression analysis. The same predictors as in the latency model were entered in block 1 and block 2. Block 1 of the regression analysis revealed possibly significant effects of *percentage of males* and *number of stimuli*. Block 2 revealed a possibly significant effect of *stimulus loudness*. These three predictors were entered into a final regression model. Table 1 lists the results. The regression model was significant for amplitude (F(3,17) = 10.317; p<.001; R^2^ = .65). Higher numbers of (summated background and target) stimuli and louder stimuli were associated with lower P300 amplitudes.

**Table 1:** Predictors from the final regression model for P300 amplitude.

	Amplitude	
	B ± SE	β
*Constant*	25.99±5.85	
*Male %*	0.11±0.06	.30
*Number of stimuli*	−0.02±0.01	−.37^a^
*Stimulus loudness*	−0.16±0.05	−.51^b^

R^2^ = .65. ^a^p<.05; ^b^p<.01.

### Cross-sectional dataset

Next, the 1,964 healthy participants from the cross-sectional dataset were used to model age-related development of the P300 latency and amplitude.

#### Psychophysiology

**Figure 4:**
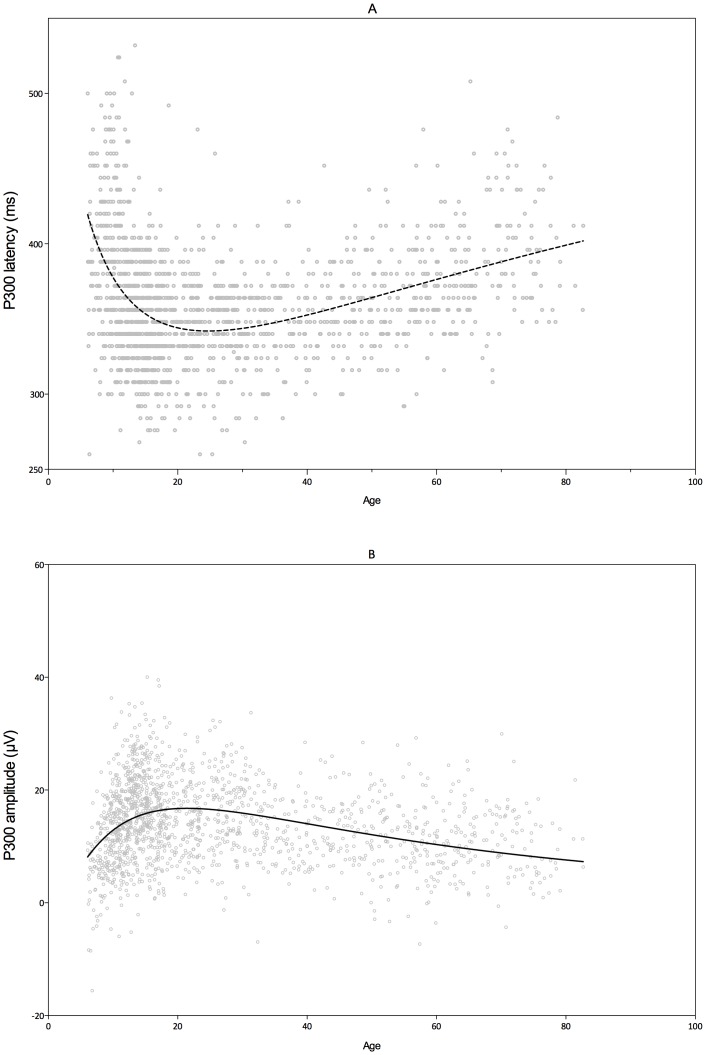
P300 latency and amplitude trajectories across the lifespan as obtained from the cross-sectional dataset. Dots represent scores from individual participants.

In figure 4a the P300 latency is plotted against age. The results of the independent cross-sectional dataset demonstrated a similar trajectory compared to the meta-analysis. P300 latency decreases during childhood, reaching a minimum in adolescence, followed by a slow increase for the rest of the lifespan. A logarithmic Gaussian model accounted for 18% of the variance and this was significantly better than a (normal) Gaussian model (F(1,1569) = 179.3; p<.0001) or a linear model (F(1,1569) = 330.6; p<.0001). In the cross-sectional dataset the minimum latency is estimated at approximately 25 years of age.

The P300 amplitude logarithmic Gaussian model is demonstrated in figure 4b. The model was able to explain 12% of the variance and this was significantly better than a (normal) Gaussian model (F(1,1569) = 108.8; p<.0001) or a linear model (F(1,1569) = 162.4; p<.0001). The maximum amplitude was reached at approximately 21 years of age.

#### Demographics

A main effect of sex was found in one-way ANOVAs on both latency (F(1,1570) = 12.606; p<.001; ω^2^ = .01) and amplitude (F(1,1570) = 10.499; p = .001; ω^2^ = .01), albeit with small effect sizes (ω^2^ = .01 is considered a small effect [44]). Separate curves for males and females demonstrated similar developmental trajectories of latency and amplitude. The curve fit statistics can be found in Table 2. None of the individual curve-fit parameters for the latency and amplitude models reached significance below the p<.01 level.

**Table 2:** p values of differences between male and female model parameters.

	Latency	Amplitude	RT
Center	.488	.032	.596
Width	.455	.306	.482
Height	.026	.053	<.001
All	.001	.001	<.001

Education effects were only investigated in a subgroup. The subgroup was defined by the high end of the respective 95% confidence intervals of centers for the latency (age is 25.5) and amplitude (age is 22.5) models until the age of 65. This group was selected since at the age of 25 most individuals will have completed their educational career. The upper cut-off was chosen to minimize a possible bias due to degenerative effects at older age. An additional advantage is that in this subgroup age-related effects on latency and amplitude can be described linearly. Regression analysis revealed no effects of education on P300 latency or amplitude in the subgroup as can be seen in Table 3.

**Table 3:** Regression analyses on latency, amplitude and reaction times.

	Latency		Amplitude		Reaction time	
	*B ± SE*	*β*	*B ± SE*	*β*	*B ± SE*	*β*
**Step 1**						
*Constant*	320.17±4.77		19.45±0.86		325.34±16.02	
*Age*	0.79±0.10	.33^b^	−0.15±0.20	−.30^b^	0.09±0.28	−.02
*Sex*	5.90±2.54	.10^a^	1.82±0.53	.138^b^	13.18±5.33	.13^a^
**Step 2**						
*Constant*	316.28±9.21		19.48±2.14		304.98±23.57	
*Age*	0.81±0.11	.33^b^	−0.16±0.02	−.31^b^	−0.03±0.28	−.01
*Sex*	5.71±2.57	.09^a^	1.71±0.53	.13^b^	−14.14±5.39	−.14^b^
*Education*	0.24±0.48	.02	−0.16±0.10	−.06	1.14±0.97	.06

Latency: R^2^ = .11 for Step 1, ΔR^2^ = .000 for Step 2 (NS). Amplitude: R^2^ = .11 for Step 1, ΔR^2^ = .004 for Step 2 (NS). RT: R^2^ = .02 for Step 1, ΔR^2^ = .004 for Step 2 (NS). ^a^ p<.05; ^b^ p<.01.

#### Behavioral measures

Reaction time was transformed by the same method used for P300 latency. A logarithmic Gaussian model was then fitted on the transformed reaction time data. The model accounted for 35% of the variance and this was significantly better than a (normal) Gaussian model (F(1,1569) = 830.2; p<.0001). The reaction time model resembles the model for P300 latency. This is confirmed by a significant partial correlation (corrected for age) between reaction time and the P300 latency (r = .30; p<.001). Pearson’s correlation coefficient indicates a medium effect size [45]. The fastest reaction times are estimated at an age of approximately 32 years of age.

A one-way ANOVA revealed a significant effect of sex (F(1,1570) = 24.26; p<.001; ω^2^ = .01), albeit with a small effect size. Separate models for male and female participants’ reaction times confirm the sex effect. These models differ significantly (F(3,1566) = 12.31; p<.0001) and show that males respond faster than females over all ages. There were no effects found for age and education in the subgroup, see table 2.

Partial correlations, correcting for age, between number of errors and P300 latencies and amplitudes were investigated. Because of the non-linear relation between age and both P300 measures the group was divided in young and older participants. Young participants were defined as all participants below the age of 20.36, which marks the low end of the 95% confidence interval for amplitude. Older participants were defined as all participants from the age of 25.54, which marks the high end of the 95% confidence interval for latency.

In young participants amplitude correlated significantly with false positive errors (r = −.106; p = .002; df = 840) and false negative errors (r = −.163; p<.001; df = 840) with small effect sizes. There were no significant correlations for latency and errors. In older participants a significant correlation was found between amplitude and false negative errors (r = −.084; p = .039; df = 601) with a small effect size.

#### Comparison of psychophysiological and behavioral trajectories

**Figure 5:**
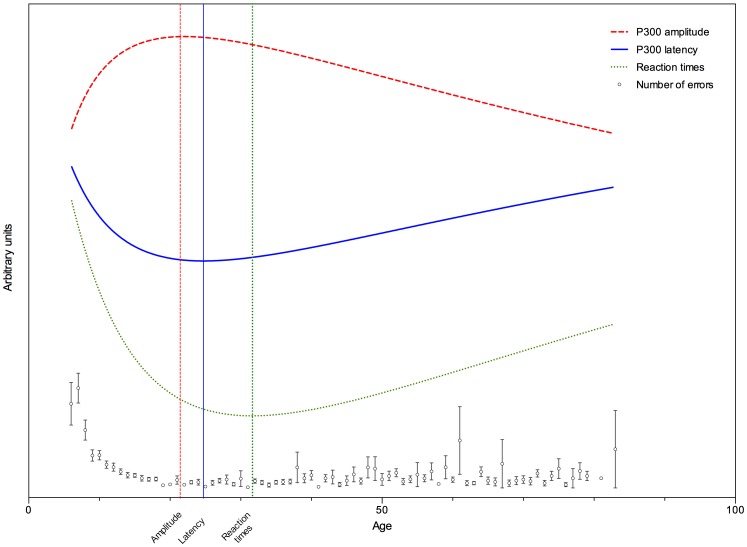
Graphical summary of the found trajectories in the cross-sectional dataset. Dots represent the number of errors. Error bars represent SEM.

The trajectories for reaction times, P300 latency and amplitude are presented in figure 5. The mean number of total errors per age are presented in the same figure. As can be seen the points of deflection (or center of the maxima and troughs) for reaction times, P300 latency and amplitude occur at different ages. These points of deflection, or model centers, were statistically compared. There was a significant difference between latency and amplitude (F1,3138) = 8.608; p = .003), as well as between latency and reaction times (F(1,3138) = 46.06; p<.0001).

## General Discussion

Developmental trajectories of the auditory P300 across the lifespan were examined using a systematic review and meta-analysis of all available literature and a large cross-sectional dataset. The P300 component was quantified in latency and amplitude measures. In both studies a logarithmic Gaussian model was the best fit for (inversed) latency and amplitude development across the lifespan. In children latency shortens until a minimum is reached. After the minimum, latency gradually increases with aging. Amplitude increases during childhood until a maximum is reached. For the rest of the lifespan amplitude decreases gradually. Sex effects were significant, but had only small effect sizes. The separate trajectories are broadly identical for males and females. In addition, education neither had an effect on latency nor amplitude. Therefore, it is concluded that the P300 *development* mainly is an endogenous process that is probably minimally influenced by exogenous factors.

The meta-analysis demonstrated that latency is not influenced by differences in paradigm parameters used. However, amplitude was affected by the number of stimuli presented and by the stimulus loudness. Specifically, a higher number of stimuli and louder stimuli were associated with smaller amplitudes. More familiar and more salient stimuli possibly required less cognitive resources, reflected by lower P300 amplitudes.

A remarkable finding in both parts of this review was that the P300 amplitude reached its maximum significantly earlier than the P300 latency reached its trough. Moreover, as found in the cross-sectional study, both latency and amplitude reached the centers of their respective models, earlier than reaction times. These findings are graphically summarized in figure 5. We therefore hypothesize that latency and amplitude index different aspects of brain maturation. The P300 latency possibly indexes neural speed or brain efficiency. The P300 amplitude might index neural power or cognitive resources, which increase with maturation.

**Figure 6:**
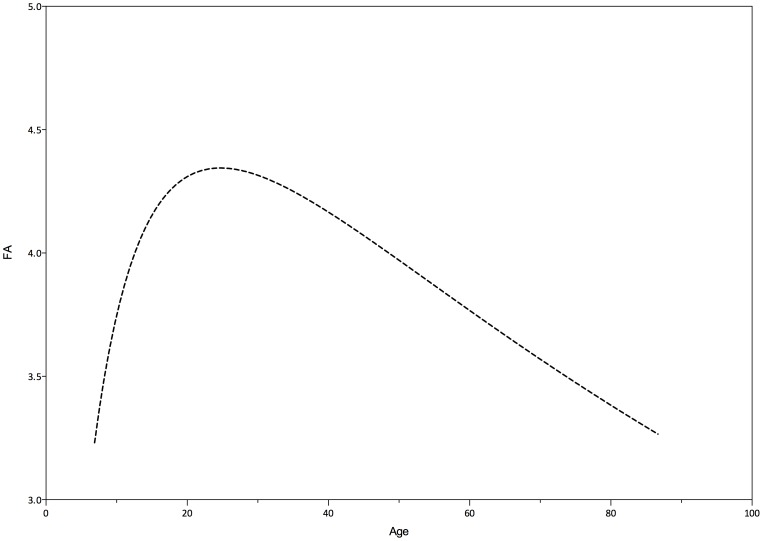
Trajectory of fractional anisotropy (FA) across the lifespan. Adapted from Brickman et(2012). Data points were estimated using DigitizeIt 1.6.1 and curve-fitted using Graphpad Prism 6.0.

As the brain develops it becomes more efficient at information processing. Structural organization and development leads to more efficient neural pathways and networks; Myelination increases neural speed. At older age increasing P300 latency is observed, which is in line with a “nearly linear decline from early adulthood on measures representing efficiency or effectiveness of processing” as described in a review by Salthouse (2010) on cognitive aging [46]. Therefore, the P300 latency might be an index for speed and efficiency of information processing in the brain. In a cross-sectional study using diffusion tensor MR imaging performed by Brickman et al. (2012), age-associated differences in measures of white matter coherence were examined in participants of 7–87 years. Figure 6 shows fractional anisotropy (FA), a diffusion tensor imaging (DTI) measure indexing myelination and organization of white matter bundles, across age. They visually inspected the DTI data plotted as a function of age to determine an approximate point of deflection at age 30 and divided their sample into two subgroups, younger and older than 30. They found white matter fiber tracts to continue developing until they reach a brief plateau, at about age 30, after which they begin degenerating [47]. The visual resemblance of their total sample with our P300 latency data is remarkable and suggests that myelination and P300 latency may be related.

As was evident from our meta-analysis, amplitude is affected by paradigm properties. Presumably, amplitude indexes the amount of cognitive resources a participant needs to allocate, to successfully perform the task at hand. In the oddball paradigm, after a participant gets more familiar with a stimulus, because the stimulus has been presented more often, fewer resources are needed to evaluate it (which is called habituation). In the same way, when a stimulus is louder, it may be easier to distinguish from the background stimuli and fewer cognitive resources are needed. As found in the cross-sectional analysis larger amplitudes are associated with fewer errors by children performing in the oddball paradigm task, reflecting more available and more allocated resources.

In the first years of life, amplitude increases when quantified using an oddball task. Although the oddball paradigm is a relatively easy task, it might still be demanding for very young children, with respect to their cognitive resources, to maintain their focus and respond accurately, which is reflected by longer reaction times and more errors. During development, children gain neural capacity and use this increased capacity to perform better on cognitive tasks. This increase in resources is quantified by an increase in amplitude until about 20 years of age.

A central question is why the P300 amplitude reaches its maximum at a younger age than the P300 latency reaches its trough? Presumably, the increase in cognitive resources and the improvement in efficiency are happening simultaneously, and the improving efficiency and neural speed might affect the model-center for P300 amplitude. A more efficient brain may not have the need to substantially recruit its cognitive capacity, if a task can be successfully performed with only a part of the available resources. Indeed, a study in adults reported that in a low demand n-back task, high performers used fewer resources by demonstrating lower P300 amplitudes in order to achieve the same performance compared to the low performers [48]. Until a certain level of efficiency is reached, a smaller proportion of cognitive resources is required to perform a given task. So, in young subjects, increasing brain efficiency and neural speed might have a moderating effect on the amplitude trajectory in which the amplitude model-center is shifted to a younger age.

As the myelination and organizational processes continue to progress after the amplitude maximum was reached and until into late adolescence, P300 latencies decline further, and task performance, quantified by shorter reaction times, improves further. The best performance is reached around the age of 30, after the optimal amplitude and latency were reached.

In older age, speed of processing is reduced [49] and behaviorally, reaction times are longer. (Subclinical) degenerative effects cause P300 latencies to increase and amplitudes to decrease. These aging-related degenerative effects are visible from neuroimaging studies as well [47], [50], where changes in white matter integrity were an important factor in executive dysfunction in older people [47]. The compensation-related utilization of neural circuits hypothesis, or ‘CRUNCH’ model states that individuals recruit additional neural activity as task load increases. The model also states that beyond a level of task demand, brains of older adults may reach their capacity limits leading to a decline in performance [48], [51]. Because of less efficient processing older people may be required to recruit additional resources at lower cognitive load levels than younger adults to achieve the same performance [52]. These compensatory mechanisms may be mediated in the prefrontal cortices [53]–[57], which is in line with the anterior shift in the topography of the P300 amplitude that has been reported in older people [58]–[61]. Therefore, it would be interesting to investigate both frontal and parietal P300 amplitude trajectories using a more cognitively challenging paradigm. Also, a distinction in the amplitude trajectories between high and low performers may provide insights into this hypothesis.

In conclusion, our findings clearly demonstrate that the P300 follows a specific trajectory across the lifespan reflecting brain maturation in childhood and adolescence and degenerative effects in older age. Although both P300 latency and amplitude can be fitted by a logarithmic Gaussian model, there are relative differences. Specifically, the centers of both models, that mark a plateau period segregating the maturation from degenerative effects, occur at different ages. This suggests that latency and amplitude reflect different aspects of brain maturation. Specifically, the P300 amplitude might be an index for the amount of cognitive resources being used, increasing in early developmental and decreasing with further aging beyond adolescence. Higher amplitudes are related to a higher proportion of allocated cognitive resources and intra-subject to improved cognitive performance. P300 latency may be a more direct index of information-processing speed and, indirectly, cognitive performance.

As far as we know, this study is the first to investigate the developmental trajectory of the P300 across the entire lifespan in a large dataset. Using advanced curve-fitting procedures we were able to determine ages that mark a maximum or trough segregating maturational from degenerative stages. The obtained trajectories are important because they provide new ways to compare healthy age-related maturation/degeneration to that associated with certain disorders (e.g. dementia, ADHD, dyslexia, schizophrenia).

There are some limitations to the current study. First, this study describes the age-related development in a large group of healthy participants. The developmental pattern that was found for this group cannot easily be translated to individual participants. There is much variation in P300 latency and amplitude between individuals, which makes it challenging to compare the P300 of a single participant to this model (albeit, this was not the primary aim of this study). Second, in the meta-analysis, some studies could not be included because they were not available to the authors, e.g. conference abstracts, old studies. Because the amount of studies to be scanned was extensive we chose to include only those studies that were available online or in local libraries. We believe the number of included studies is sufficient by this method, also evidenced by the similarities in results as compared to the cross-sectional sample. Although the results would have been stronger if more studies could have been included, we do not expect the results would be different from the ones presented in this paper. Third, the reported P300 latencies and amplitudes were measured using a peak-picking method. The highest peak of a component in the ERP is arbitrary since it does not represent any meaningful information about this component. Although this method is very conventional, especially in older studies, its validity can be questioned. This problem is partly solved by the large datasets that were used in our study.

## Supporting Information

Table S1 **Overview of all the studies that were included in the meta-analysis.** Studies marked with an asterisk (*) reported data for subgroups that were included as such.
(DOCX)

Checklist S1 **PRISMA Checklist.**
(DOC)
