# Supplementary material for: P300 Development across the Lifespan: A Systematic Review and Meta-Analysis
Source: PLoS One. 2014 Feb 13;9(2):e87347. doi: 10.1371/journal.pone.0087347 (PMC3923761; doi:10.1371/journal.pone.0087347)
Supplement: Table S1 — Overview of all the studies that were included in the meta-analysis. Studies marked with an asterisk (*) reported data for subgroups that were included as such. (DOCX) [file pone.0087347.s001.docx]

# Table S1

Overview of studies that were included in the meta-analysis

| Study | N | Male percentage | Mean age (years) | Age range (years) | Continent | Target probability (%) | Eyes open / closed | Reponse type | Total number of stimuli | Stimulus duration (ms) | Stimulus loudness (dB SPL) |
| --- | --- | --- | --- | --- | --- | --- | --- | --- | --- | --- | --- |
| [1]* | 55 | 49 | 49.8 | 15-89 | Australia | 15 | - | count | 400 | 50 | 80 |
| [2]* | 12 | - | 56.6 | 28-81 | North America | 20 | open | press | 300 | 50 | 72 |
| [3] | 32 | 88 | 20.6 | 18-24 | Asia | 20 | - | count | - | 90 | 75 |
| [4]* | 63 | 43 | 42.9 | 22-78 | Asia | 15 | - | press | - | - | 75 |
| [5] | 21 | - | 34.0 | 22-55 | Europe | 20 | - | press | - | 40 | 64 |
| [6]* | 154 | - | 9.7 | 4-15 | Asia | 20 | closed | press | 160 | 120 | 60 |
| [7] | 20 | - | 64.1 | 43-80 | Europe | 33 | - | count | 200 | 20 | - |
| [8] | 20 | 50 | 39.3 | 25-59 | Asia | 25 | closed | press | 160 | 100 | 75 |
| [9] | 16 | 69 | 31.9 | - | Europe | 20 | closed | count | 150 | 40 | 97 |
| [10] | 28 | 64 | 31.2 | - | Europe | 15 | - | count | - | 40 | 75 |
| [11] | 29 | 100 | 11.5 | - | North America | 10 | closed | count | 264 | 50 | 64.5 |
| [12] | 78 | 40 | 28.6 | - | Asia | 20 | - | press | - | - | - |
| [13] | 19 | 84 | 28.6 | - | Europe | 20 | closed | press | 500 | 40 | 80 |
| [14]* | 72 | 50 | 10.1 | 6-8 | Europe | 20 | closed | press | - | 50 | 60 |
| [15]* | 18 | - | 29.3 | - | Europe | 20 | closed | count | 150 | 40 | 97 |
| [16] | 36 | 61 | 27.5 | - | Europe | 15 | - | count | 130 | 50 | - |
| [17] | 41 | 100 | 24.5 | 20-33 | North America | 12.5 | open | press | - | 60 | 60 |
| [18] | 21 | 62 | 28.9 | - | Europe | 20 | - | press | - | - | - |
| [19] | 23 | 65 | 30.6 | - | North America | 20 | open | press | - | 50 | 60 |
| [20] | 15 | 73 | 43.7 | 28-70 | North America | 15 | - | press | 600 | 40 | - |
| [21] | 29 | 62 | 66.7 | 46-79 | Europe | 20 | open | press | - | 100 | - |
| [22] | 19 | 37 | 26.8 | - | Europe | 20 | - | press | 200 | 100 | 65 |
| [23] | 40 | 73 | 36.7 | 20-54 | Australia | 15 | open | press | 287 | 50 | 60 |
| [24] | 39 | 46 | 68.5 | 60-77 | Asia | 20 | - | press | - | 50 | 70 |
| [25] | 28 | 100 | 29.0 | 18-45 | Europe | 20 | open | press | - | 120 | - |
| [26] | 55 | 52 | 60.5 | 40-79 | Asia | 20 | - | count | 400 | 50 | - |
| [27] | 15 | 100 | 27.0 | - | Asia | 20 | open | press | - | 120 | - |
| [28]* | 32 | 56 | 31.0 | 10-68 | Europe | 20 | closed | count | 100 | 120 | 60 |
| [29]* | 72 | 50 | 57.6 | 22-95 | Europe | 20 | - | count | 240 | 80 | - |
| [30] | 18 | 67 | 15.5 | - | Asia | 20 | closed | press | 200 | 200 | 90 |
| [31] | 17 | 35 | 37.9 | - | Australia | 15 | open | press | 287 | 50 | 60 |
| [32] | 22 | 45 | 41.4 | - | Europe | 15 | - | press | - | 100 | - |
| [33]* | 80 | 68 | 28.2 | 14-51 | Australia | 15 | open | press | 287 | 50 | 60 |
| [34]* | 59 | 47 | 56.1 | 22-92 | Europe | 20 | - | count | - | 80 | 80 |
| [35] | 33 | 45 | 27.8 | - | Asia | 15 | - | press | 270 | 50 | 75 |
| [36] | 25 | 64 | 28.9 | - | Asia | 15 | - | count | 400 | 200 | - |
| [37] | 36 | 58 | 25.8 | 20-37 | Asia | 20 | - | press | - | 100 | 70 |
| [38] | 18 | 56 | 26.0 | 22-34 | Europe | 20 | - | count | 240 | 80 | 80 |
| [39] | 28 | 79 | 24.0 | 18-47 | North America | 15 | - | press | 200 | 50 | 97 |
| [40] | 23 | 100 | 9.2 | 7-12 | Europe | 20 | closed | count | - | - | - |
| [41] | 16 | 56 | 11.9 | 10-13 | Europe | 20 | open | count | - | 100 | - |
| [42] | 20 | 50 | 25.0 | 19-31 | Europe | 20 | - | press | 400 | 60 | - |
| [43]* | 16 | 50 | 26.5 | 20-34 | Europe | 20 | - | press | - | - | - |
| [44] | 21 | 52 | 7.0 | - | Europe | 20 | closed | press | 238 | 400 | - |
| [45] | 15 | 53 | 34.7 | 23-55 | Europe | 20 | - | count | - | 50 | - |
| [46] | 53 | 62 | 73.6 | - | Europe | 20 | closed | press | 100 | 100 | 80 |
| [47]* | 40 | 45 | 64.6 | 39-89 | North America | 15 | - | count | - | 50 | 80 |
| [48] | 16 | 81 | 67.6 | - | Europe | 20 | - | count | - | 100 | 75 |
| [49] | 40 | 55 | 25.7 | 20-36 | Asia | 20 | closed | press | - | 100 | 70 |
| [50]* | 192 | - | 46.0 | 20-65 | Europe | 25 | closed | count | 512 | 50 | 70 |
| [51] | 35 | 69 | 29.3 | - | Europe | 14 | - | press | - | 50 | - |
| [52] | 15 | 100 | 21.3 | 16-25 | Asia | 20 | closed | press | - | 50 | - |
| [53] | 36 | 42 | 17.2 | 14-21 | Europe | 15 | open | press | 300 | 50 | - |
| [54] | 42 | 48 | 40.2 | 18-60 | Europe | 20 | - | press | 400 | 20 | - |
| [55] | 57 | 42 | 24.5 | 19-35 | Europe | 20 | open | press | 400 | - | - |
| [56] | 26 | 0 | 30.9 | - | Asia | 20 | closed | press | - | 100 | - |
| [57]* | 68 | 50 | 50.0 | 20-80 | Europe | 20 | - | press | 400 | 50 | 110 |
| [58] | 15 | 47 | 34.3 | - | North America | 15 | closed | count | 385 | - | 110 |
| [59] | 16 | 44 | 74.0 | 60-92 | Europe | 20 | closed | press | 200 | 80 | 75 |
| [60] | 28 | 82 | 21.7 | 17-27 | Europe | 20 | - | press | 300 | 100 | 75 |
| [61] | 40 | 60 | 25.7 | 18-39 | Europe | 10 | closed | press | 384 | 500 | - |
| [62] | 20 | 60 | 69.5 |  | Europe | 15 | - | press | 213 | 100 | - |
| [63] | 32 | 38 | 23.4 | 19-34 | Europe | 20 | - | count | - | 50 | 65 |
| [64] | 38 | 100 | 49.0 | 25-58 | Asia | 20 | closed | count | 250 | 120 | - |
| [65]* | 57 | 47 | 13.0 | 7-18 | Asia | 20 | closed | count | 100 | 50 | - |
| [66] | 17 | 53 | 23.9 | - | Asia | 25 | open | press | - | 70 | - |
| [67] | 25 | 48 | 32.9 | 17-52 | Asia | 20 | - | press | 250 | 50 | 80 |
| [68] | 110 | 33 | 11.3 | 10.2-12.9 | North America | 20 | open | press | 400 | 50 | - |
| [69] | 17 | 71 | 57.2 | 27-81 | Europe | - | open | count | 240 | 400 | - |
| [70] | 40 | 38 | 25.6 | 18-45 | Asia | 16.7 | - | count | - | 50 | - |
| [71] | 17 | 65 | 20.1 | - | Europe | 20 | open | unknown / subjects choice | 300 | 100 | - |
| [72] | 23 | 57 | 10.3 | - | Europe | 20 | closed | count | - | - | - |
| [73] | 33 | 61 | 32.4 | - | Asia | 15 | - | press | 400 | 100 | 85 |
| [74] | 27 | 59 | 51.1 | 20-72 | Europe | 20 | - | press | - | 60 | - |
| [75] | 51 | 78 | 9.0 | 6-13 | Asia | 20 | closed | unknown / subjects choice | 200 | - | - |

* This study reported data for subgroups that were used in the meta-analysis.

# References

1 Kraiuhin C, Gordon E, Stanfield P, Meares R, Howson A (1986) P300 and the effects of aging: relevance to the diagnosis of dementia. Exp Aging Res 12: 187-192. doi: 10.1080/03610738608258566

2 Patterson JV, Michalewski HJ, Starr A (1988) Latency variability of the components of auditory event-related potentials to infrequent stimuli in aging, Alzheimer-type dementia, and depression. Electroencephalogr Clin Neurophysiol 71: 450-460.

3 Ogura C, Hirano K, Nageishi Y, Takeshita S, Fukao K, et al (1994) Deviate P200 and P300 in non-patient college students with high scores on the schizophrenia scale of the Minnesota Multiphasic Personality Inventory (MMPI). Int J Psychophysiol 16: 89-97.

4 Kakigi R, Neshige R, Matsuda Y, Kuroda Y (1994) Auditory P300 response in Down's syndrome: comparison with Alzheimer-type dementia and normal controls. Pathophysiology 1: 35-39.

5 Wang W, Schoenen J, Timsit-Berthier M (1995) Cognitive functions in migraine without aura between attacks: a psychophysiological approach using the "oddball" paradigm. Neurophysiol Clin 25: 3-11. doi: 10.1016/0987-7053(96)81029-X

6 Fuchigami T, Okubo O, Ejiri K, Fujita Y, Kohira R, et al (1995) Developmental changes in P300 wave elicited during two different experimental conditions. Pediatr Neurol 13: 25-28.

7 Gil R, Neau JP, Dary-Auriol M, Agbo C, Tantot AM, Ingrand P (1995) Event-related auditory evoked potentials and amyotrophic lateral sclerosis. Arch Neurol 52: 890-896.

8 Iwanami A, Kamijima K, Yoshizawa J (1996) P300 component of event-related potentials in passive tasks. Int J Neurosci 84: 121-126.

9 Weisbrod M, Winkler S, Maier S, Hill H, Thomas C, Spitzer M (1997) Left lateralized P300 amplitude deficit in schizophrenic patients depends on pitch disparity. Biol Psychiatry 41: 541-549.

10 Shajahan PM, O'Carroll RE, Glabus MF, Ebmeier KP, Blackwood DH (1997) Correlation of auditory 'oddball' P300 with verbal memory deficits in schizophrenia. Psychol Med 27: 579-586.

11 Brigham J, Moss HB, Murrelle EL, Kirisci L, Spinelli JS (1997) Event-related potential negative shift in sons of polysubstance- and alcohol-use disorder fathers. Psychiatry Res 73: 133-146.

12 Kubota F, Kifune A, Shibata N, Akata T, Takeuchi K, Takahashi S (1998) Study on the P300 of adult epileptic patients (unmedicated and medicated patients). Journal of Epilepsy 11: 325 - 331. doi: 10.1016/S0896-6974(98)00040-1

13 Frodl-Bauch T, Gallinat J, Meisenzahl EM, Möller HJ, Hegerl U (1999) P300 subcomponents reflect different aspects of psychopathology in schizophrenia. Biol Psychiatry 45: 116-126.

14 Zenker F, Barajas JJ (1999) Auditory P300 development from an active, passive and single-tone paradigms. Int J Psychophysiol 33: 99-111.

15 Weisbrod M, Hill H, Niethammer R, Sauer H (1999) Genetic influence on auditory information processing in schizophrenia: P300 in monozygotic twins. Biol Psychiatry 46: 721-725.

16 Pallanti S, Quercioli L, Pazzagli A (1999) Basic symptoms and P300 abnormalities in young schizophrenic patients. Compr Psychiatry 40: 363-371.

17 Ji J, Porjesz B, Begleiter H, Chorlian D (1999) P300: the similarities and differences in the scalp distribution of visual and auditory modality. Brain Topogr 11: 315-327.

18 Karoumi B, Laurent A, Rosenfeld F, Rochet T, Brunon AM, et al (2000) Alteration of event related potentials in siblings discordant for schizophrenia. Schizophr Res 41: 325-334.

19 Turetsky BI, Cannon TD, Gur RE (2000) P300 subcomponent abnormalities in schizophrenia: III. Deficits In unaffected siblings of schizophrenic probands. Biol Psychiatry 47: 380-390.

20 Kimble M, Lyons M, O'Donnell B, Nestor P, Niznikiewicz M, Toomey R (2000) The effect of family status and schizotypy on electrophysiologic measures of attention and semantic processing. Biol Psychiatry 47: 402-412.

21 Korpelainen JT, Kauhanen ML, Tolonen U, Brusin E, Mononen H, et al (2000) Auditory P300 event related potential in minor ischemic stroke. Acta Neurol Scand 101: 202-208.

22 Pierson A, Jouvent R, Quintin P, Perez-Diaz F, Leboyer M (2000) Information processing deficits in relatives of manic depressive patients. Psychol Med 30: 545-555.

23 Brown K, Gordon E, Williams L, Bahramali H, Harris A, et al (2000) Misattribution of sensory input reflected in dysfunctional target:non-target ERPs in schizophrenia. Psychol Med 30: 1443-1449.

24 Sumi N, Nan'no H, Fujimoto O, Ohta Y, Takeda M (2000) Interpeak latency of auditory event-related potentials (P300) in senile depression and dementia of the Alzheimer type. Psychiatry Clin Neurosci 54: 679-684. doi: 10.1046/j.1440-1819.2000.00769.x

25 Bond AJ, Surguy SM (2000) Relationship between attitudinal hostility and P300 latencies. Prog Neuropsychopharmacol Biol Psychiatry 24: 1277-1288.

26 Iijima M, Osawa M, Iwata M, Miyazaki A, Tei H (2000) Topographic mapping of P300 and frontal cognitive function in Parkinson's disease. Behav Neurol 12: 143-148.

27 Jing H, Takigawa M, Hamada K, Okamura H, Kawaika Y, et al (2001) Effects of high frequency repetitive transcranial magnetic stimulation on P(300) event-related potentials. Clin Neurophysiol 112: 304-313.

28 Caravaglios G, Natalè E, Ferraro G, Fierro B, Raspanti G, Daniele O (2001) Auditory event-related potentials (P300) in epileptic patients. Neurophysiol Clin 31: 121-129.

29 Fjell AM, Walhovd KB (2001) P300 and neuropsychological tests as measures of aging: scalp topography and cognitive changes. Brain Topogr 14: 25-40.

30 Kim MS, Kim JJ, Kwon JS (2001) Frontal P300 decrement and executive dysfunction in adolescents with conduct problems. Child Psychiatry Hum Dev 32: 93-106.

31 Felmingham KL, Bryant RA, Kendall C, Gordon E (2002) Event-related potential dysfunction in posttraumatic stress disorder: the role of numbing. Psychiatry Res 109: 171 - 179. doi: 10.1016/S0165-1781(02)00003-3

32 Vuurman EFPM, Honig A, Lamers H, Wiersma J, Krabbendam L, et al (2002) Event-related potentials and white matter lesions in bipolar disorder. Acta Neuropsychiatrica 14: 11-16. doi: 10.1034/j.1601-5215.2002.140102.x

33 Brown KJ, Gonsalvez CJ, Harris AW, Williams LM, Gordon E (2002) Target and non-target ERP disturbances in first episode vs. chronic schizophrenia. Clin Neurophysiol 113: 1754-1763.

34 Walhovd KB, Fjell AM (2002) One-year test–retest reliability of auditory ERPs in young and old adults. Int J Psychophysiol 46: 29 - 40. doi: 10.1016/S0167-8760(02)00039-9

35 Iwanami A, Kato N, Kasai K, Kamio S, Furukawa S, et al (2002) P300 amplitude over temporal regions in schizophrenia. Eur Arch Psychiatry Clin Neurosci 252: 1-7.

36 Kim MS, Cho SS, Kang KW, Hwang JL, Kwon JS (2002) Electrophysiological correlates of personality dimensions measured by Temperament and Character Inventory. Psychiatry Clin Neurosci 56: 631-635. doi: 10.1046/j.1440-1819.2002.01067.x

37 Higashima M, Nagasawa T, Kawasaki Y, Oka T, Sakai N, et al (2003) Auditory P300 amplitude as a state marker for positive symptoms in schizophrenia: cross-sectional and retrospective longitudinal studies. Schizophr Res 59: 147-157.

38 Fjell AM, Walhovd KB (2003) Effects of auditory stimulus intensity and hearing threshold on the relationship among P300, age, and cognitive function. Clin Neurophysiol 114: 799-807.

39 Salisbury DF, Griggs CB, Shenton ME, McCarley RW (2004) The NoGo P300 'anteriorization' effect and response inhibition. Clin Neurophysiol 115: 1550-1558. doi: 10.1016/j.clinph.2004.01.028

40 Ozdag MF, Yorbik O, Ulas UH, Hamamcioglu K, Vural O (2004) Effect of methylphenidate on auditory event related potential in boys with attention deficit hyperactivity disorder. Int J Pediatr Otorhinolaryngol 68: 1267-1272. doi: 10.1016/j.ijporl.2004.04.023

41 Karlidag R, Ozisik HI, Soylu A, Kizkin S, Sipahi B, et al (2004) Topographic abnormalities in event-related potentials in children with monosymptomatic nocturnal enuresis. Neurourol Urodyn 23: 237-240. doi: 10.1002/nau.20031

42 Lalo E, Vercueil L, Bougerol T, Jouk PS, Debû B (2005) Late event-related potentials and movement complexity in young adults with Down syndrome. Neurophysiol Clin 35: 81-91. doi: 10.1016/j.neucli.2005.03.002

43 Szinnai G, Schachinger H, Arnaud MJ, Linder L, Keller U (2005) Effect of water deprivation on cognitive-motor performance in healthy men and women. Am J Physiol Regul Integr Comp Physiol 289: R275-R280. doi: 10.1152/ajpregu.00501.2004

44 Huber M, Telser S, Falk M, Böhm A, Hackenberg B, et al (2005) Information transmission defect identified and localized in language learning impaired children by means of electrophysiology. Cortex 41: 464-470.

45 Korostenskaja M, Dapsys K, Siurkute A, Maciulis V, Ruksenas O, Kähkönen S (2005) Effects of olanzapine on auditory P300 and mismatch negativity (MMN) in schizophrenia spectrum disorders. Prog Neuropsychopharmacol Biol Psychiatry 29: 543-548. doi: 10.1016/j.pnpbp.2005.01.019

46 van Harten B, Laman DM, van Duijn H, Knol DL, Stam CJ, et al (2006) The auditory oddball paradigm in patients with vascular cognitive impairment: a prolonged latency of the N2 complex. Dement Geriatr Cogn Disord 21: 322-327. doi: 10.1159/000091474

47 Ally BA, Jones GE, Cole JA, Budson AE (2006) The P300 component in patients with Alzheimer's disease and their biological children. Biol Psychol 72: 180-187. doi: 10.1016/j.biopsycho.2005.10.004

48 Raggi A, Manconi M, Consonni M, Martinelli C, Zucconi M, et al (2007) Event-related potentials in idiopathic rapid eye movements sleep behaviour disorder. Clin Neurophysiol 118: 669-675. doi: 10.1016/j.clinph.2006.11.011

49 Higashima M, Tsukada T, Nagasawa T, Oka T, Okamoto T, et al (2007) Reduction in event-related alpha attenuation during performance of an auditory oddball task in schizophrenia. Int J Psychophysiol 65: 95-102. doi: 10.1016/j.ijpsycho.2007.03.008

50 Keski-Säntti P, Holm A, Akila R, Tuisku K, Kovala T, Sainio M (2007) P300 of auditory event related potentials in occupational chronic solvent encephalopathy. Neurotoxicology 28: 1230-1236. doi: 10.1016/j.neuro.2007.08.004

51 Müller BW, Specka M, Steinchen N, Zerbin D, Lodemann E, et al (2007) Auditory target processing in methadone substituted opiate addicts: the effect of nicotine in controls. BMC Psychiatry 7: 63. doi: 10.1186/1471-244X-7-63

52 Lebedeva IS, Kaleda VG, Barkhatova AN (2008) Neurophysiological characteristics of cognitive functions in patients with first episodes of endogenous psychosis. Neurosci Behav Physiol 38: 259-267. doi: 10.1007/s11055-008-0038-7

53 Groom MJ, Bates AT, Jackson GM, Calton TG, Liddle PF, Hollis C (2008) Event-related potentials in adolescents with schizophrenia and their siblings: a comparison with attention-deficit/hyperactivity disorder. Biol Psychiatry 63: 784-792. doi: 10.1016/j.biopsych.2007.09.018

54 Schulze KK, Hall MH, McDonald C, Marshall N, Walshe M, et al (2008) Auditory P300 in patients with bipolar disorder and their unaffected relatives. Bipolar Disord 10: 377-386. doi: 10.1111/j.1399-5618.2007.00527.x

55 Bramon E, Shaikh M, Broome M, Lappin J, Bergé D, et al (2008) Abnormal P300 in people with high risk of developing psychosis. Neuroimage 41: 553-560. doi: 10.1016/j.neuroimage.2007.12.038

56 Anjana Y, Tandon OP, Vaney N, Madhu SV (2008) Cognitive status in hypothyroid female patients: event-related evoked potential study. Neuroendocrinology 88: 59-66. doi: 10.1159/000117713

57 Schiff S, Valenti P, Andrea P, Lot M, Bisiacchi P, et al (2008) The effect of aging on auditory components of event-related brain potentials. Clin Neurophysiol 119: 1795-1802. doi: 10.1016/j.clinph.2008.04.007

58 Gooding DC, Burroughs S, Boutros NN (2008) Attentional deficits in cocaine-dependent patients: converging behavioral and electrophysiological evidence. Psychiatry Res 160: 145-154. doi: 10.1016/j.psychres.2007.11.019

59 Caravaglios G, Costanzo E, Palermo F, Muscoso EG (2008) Decreased amplitude of auditory event-related delta responses in Alzheimer's disease. Int J Psychophysiol 70: 23-32. doi: 10.1016/j.ijpsycho.2008.04.004

60 de Wilde OM, Bour LJ, Dingemans PM, Koelman JH, Boerée T, Linszen DH (2008) P300 deficits are present in young first-episode patients with schizophrenia and not in their healthy young siblings. Clin Neurophysiol 119: 2721-2726. doi: 10.1016/j.clinph.2008.08.024

61 Frommann I, Brinkmeyer J, Ruhrmann S, Hack E, Brockhaus-Dumke A, et al (2008) Auditory P300 in individuals clinically at risk for psychosis. Int J Psychophysiol 70: 192-205. doi: 10.1016/j.ijpsycho.2008.07.003

62 van Deursen JA, Vuurman EF, Smits LL, Verhey FR, Riedel WJ (2009) Response speed, contingent negative variation and P300 in Alzheimer's disease and MCI. Brain Cogn 69: 592-599. doi: 10.1016/j.bandc.2008.12.007

63 Karakaş HM, Karakaş S, Ozkan Ceylan A, Tali ET (2009) Recording event-related activity under hostile magnetic resonance environment: Is multimodal EEG/ERP-MRI recording possible? Int J Psychophysiol 73: 123-132. doi: 10.1016/j.ijpsycho.2009.03.006

64 Dassanayake T, Gawarammana IB, Weerasinghe V, Dissanayake PS, Pragaash S, et al (2009) Auditory event-related potential changes in chronic occupational exposure to organophosphate pesticides. Clin Neurophysiol 120: 1693-1698. doi: 10.1016/j.clinph.2009.07.034

65 Rozhkov VP, Sergeeva EG, Soroko SI (2009) Age dynamics of evoked brain potentials in involuntary and voluntary attention to a deviant stimulus in schoolchildren from the northern region. Neurosci Behav Physiol 39: 851-863. doi: 10.1007/s11055-009-9210-y

66 Choi JW, Jung KY, Kim CH, Kim KH (2010) Changes in gamma- and theta-band phase synchronization patterns due to the difficulty of auditory oddball task. Neurosci Lett 468: 156-160. doi: 10.1016/j.neulet.2009.10.088

67 Wang J, Tang Y, Li C, Mecklinger A, Xiao Z, et al (2010) Decreased P300 current source density in drug-naive first episode schizophrenics revealed by high density recording. Int J Psychophysiol 75: 249-257. doi: 10.1016/j.ijpsycho.2009.12.005

68 Boucher O, Bastien CH, Muckle G, Saint-Amour D, Jacobson SW, Jacobson JL (2010) Behavioural correlates of the P3b event-related potential in school-age children. Int J Psychophysiol 76: 148-157. doi: 10.1016/j.ijpsycho.2010.03.005

69 Volpato C, Piccione F, Silvoni S, Cavinato M, Palmieri A, et al (2010) Working memory in amyotrophic lateral sclerosis: auditory event-related potentials and neuropsychological evidence. J Clin Neurophysiol 27: 198-206. doi: 10.1097/WNP.0b013e3181e0aa14

70 Huang MW, Chou FH, Lo PY, Cheng KS (2011) A comparative study on long-term evoked auditory and visual potential responses between Schizophrenic patients and normal subjects. BMC Psychiatry 11: 74. doi: 10.1186/1471-244X-11-74

71 van Tricht MJ, Nieman DH, Koelman JH, Bour LJ, van der Meer JN, et al (2011) Auditory ERP components before and after transition to a first psychotic episode. Biol Psychol 87: 350-357. doi: 10.1016/j.biopsycho.2011.04.005

72 Tascilar ME, Turkkahraman D, Oz O, Yucel M, Taskesen M, et al (2011) P300 auditory event-related potentials in children with obesity: is childhood obesity related to impairment in cognitive functions? Pediatr Diabetes 12: 589-595. doi: 10.1111/j.1399-5448.2010.00748.x

73 Bae KY, Kim DW, Im CH, Lee SH (2011) Source imaging of P300 auditory evoked potentials and clinical correlations in patients with posttraumatic stress disorder. Prog Neuropsychopharmacol Biol Psychiatry 35: 1908-1917. doi: 10.1016/j.pnpbp.2011.08.002

74 Balaban H, Şentürk IA, Yildiz ÖK, Bolayir E, Topaktaş S (2012) The role of event-related potentials in subclinical cognitive dysfunction in essential tremor. J Clin Neurophysiol 29: 65-69. doi: 10.1097/WNP.0b013e318246ad89

75 Tsai ML, Hung KL, Lu HH (2012) Auditory event-related potentials in children with attention deficit hyperactivity disorder. Pediatr Neonatol 53: 118-124. doi: 10.1016/j.pedneo.2012.01.009
